# Imprinting defects at human 14q32 locus alters gene expression and is associated with the pathobiology of osteosarcoma

**DOI:** 10.18632/oncotarget.6965

**Published:** 2016-01-21

**Authors:** Jingmin Shu, Lihua Li, Anne E. Sarver, Emily A. Pope, Jyotika Varshney, Venugopal Thayanithy, Logan Spector, David A. Largaespada, Clifford J. Steer, Subbaya Subramanian

**Affiliations:** ^1^ Masonic Cancer Research Center, University of Minnesota, Minneapolis, MN, USA; ^2^ Department of Surgery, University of Minnesota, Minneapolis, MN, USA; ^3^ Department of Genetics, Cell Biology and Development, University of Minnesota, Minneapolis, MN, USA; ^4^ Department of Pediatrics, University of Minnesota, Minneapolis, MN, USA; ^5^ Department of Medicine, University of Minnesota, Minneapolis, MN, USA

**Keywords:** osteosarcoma, 14q32-locus, imprinting, DNA methylation, histone modifications

## Abstract

Osteosarcoma is the most common primary bone malignancy affecting children and adolescents. Although several genetic predisposing conditions have been associated with osteosarcoma, our understanding of its pathobiology is rather limited. Here we show that, first, an imprinting defect at human 14q32-locus is highly prevalent (87%) and specifically associated with osteosarcoma patients < 30 years of age. Second, the average demethylation at differentially methylated regions (DMRs) in the 14q32-locus varied significantly compared to genome-wide demethylation. Third, the 14q32-locus was enriched in both H3K4-me3 and H3K27-me3 histone modifications that affected expression of all imprinted genes and miRNAs in this region. Fourth, imprinting defects at 14q32 - DMRs are present in triad DNA samples from affected children and their biological parents. Finally, imprinting defects at 14q32-DMRs were also observed at higher frequencies in an *Rb1/Trp53* mutation-induced osteosarcoma mouse model. Further analysis of normal and tumor tissues from a *Sleeping Beauty* mouse model of spontaneous osteosarcoma supported the notion that these imprinting defects may be a key factor in osteosarcoma pathobiology. In conclusion, we demonstrate that imprinting defects at the 14q32 locus significantly alter gene expression, may contribute to the pathogenesis of osteosarcoma, and could be predictive of survival outcomes.

## INTRODUCTION

Osteosarcoma is an aggressive bone cancer predominantly affecting children and young adolescents with a 5-year survival rate of ~70% [1]. Despite significant advances in cancer treatment, the survival rate for osteosarcoma has remained static for the past 30 years. Genetic deficiencies such as mutations of *TP53, RB, p14* and *p16* have previously been reported as occurring frequently in osteosarcoma [2–5]. Silencing of Wnt inhibitory factor 1(WIF1) [6], p14ARF [7], and RASSF1A [8] by DNA hypermethylation and loss-of-imprinting in IGF2 and H19 have also been shown to occur in osteosarcoma [9, 10]. Recently, we demonstrated that microRNAs (miRNAs) at the14q32 locus are significantly downregulated in osteosarcoma, but not due to a loss of DNA copy number [11]. The 14q32 imprinted region is ~ 1 Mb in size and contains both paternally (*DLK1*, *RTL1* and *DIO3*) and maternally (*MEG3*, *MEG8* and *DIO3OS*) expressed genes [12, 13]. These imprinted genes have key roles in cellular functions [14–16], bone differentiation [17, 18], and can serve as markers for complete reprogramming of induced pluripotent stem (iPS) cells [19]. Interestingly, this imprinted locus also includes over 40 miRNAs, a subset of which cooperatively regulates cMYC expression in osteosarcoma [11]. It is therefore likely that dysregulated expression of genes and miRNAs resulting from disordered imprinting at this locus could contribute to the pathogenesis of osteosarcoma [20, 21].

Genomic imprinting is a type of epigenetic inheritance through which differential expression of genes depends on parental origin (i.e., maternal or paternal) [22, 23]. Typically, genomic imprinting occurs in pairs of genes that are located near each other. The expression of these imprinted genes is controlled by DNA methylation of unique parent-origin-alleles in the imprinting control regions (ICRs) or differentially methylated regions (DMRs) [24]. Loss of the monoallelic DNA methylation pattern in the ICRs or DMRs, also known as loss-of-imprinting, can cause changes in expression of imprinted genes in the region. For example, loss-of-imprinting at the *IGF2* locus has been found in Wilms' tumor [25, 26], colorectal [27], and other cancer types [28]. The three DMRs in the 14q32 locus can regulate the imprinted genes and miRNA clusters in the region [29, 30].

To determine if epigenetic alteration(s) of the 14q32 locus is associated with osteosarcoma, we evaluated the DNA methylation levels of these DMRs and studied histone modifications in normal bone tissues and osteosarcoma patient samples. We also determined the imprinting status of the 14q32 locus in buccal DNA samples from osteosarcoma patients and their biological parents. Further, we studied the methylation patterns at DMRs of the homologous regions in two mouse models of spontaneous osteosarcoma. Our results suggest that imprinting defects in humans is associated with the pathobiology of osteosarcoma.

## RESULTS

### Imprinting defects at 14q32 DMRs in osteosarcoma

**Figure 1:**
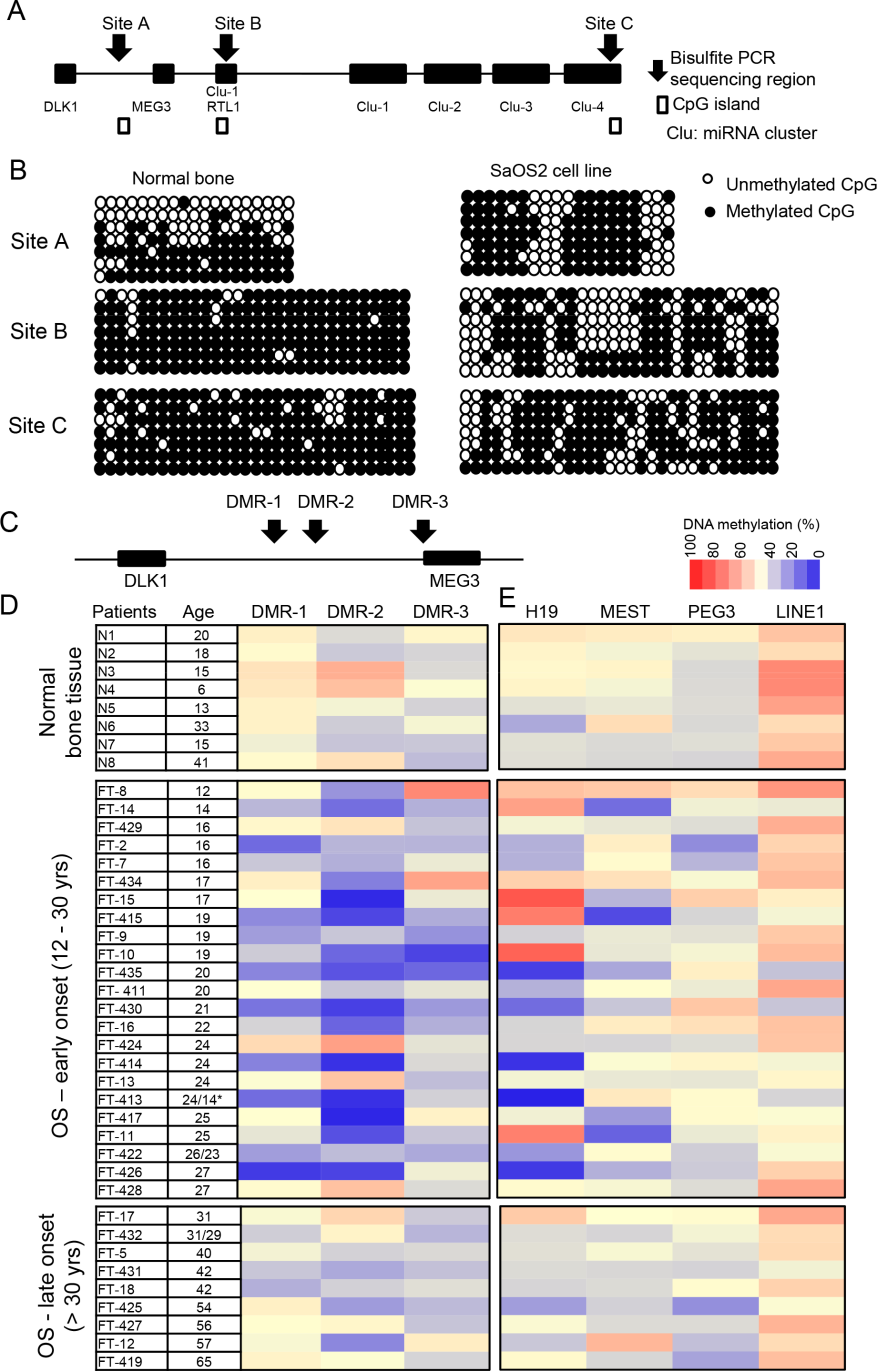
Confirmation of IG-DMR of 14q32 imprinted locus and DNA methylation changes of DMRs at the 14q32-locus (**A**) Schematic map of the 14q32 imprinted locus. DNA methylation levels of sites A, B, and C were studied by bisulfite TA cloning and sequencing. (**B**) DNA methylation pattern of sites A, B and C in normal bone tissue and SaOS2 cell line. Site A includes DMR2 in Figure 1C. Each row represents a single clone and each column represents a CpG site. (**C**) Three DMRs at 14q32-locus. DMR-1 and DMR-2 are IG- DMRs, and DMR-3 is MEG3-DMR. (**D**) DNA methylation levels of DMRs of 14q32-locus, 8 normal bone tissue and 32 osteosarcoma patient tumor tissue samples analyzed by bisulfite pyrosequencing. (**E**) DNA methylation levels of DMRs of three other imprinted loci, including H19, MEST and PEG3, and methylation levels of LINE1. *Age: 24/14 represent tissues from relapse osteosarcoma samples (24 years) and primary at 14 years.

There are three DMRs in the 14q32-imprinted region, which can regulate the entire imprinted gene cluster [29]. Two of these DMRs, DMR-1 and DMR- 2, are intergenic (IG-DMRs) and are located in close proximity to each other between the *DLK1* and *MEG3* genes. The third, DMR-3, is located in the promoter region of the *MEG3* gene (MEG3-DMR). By bisulfite TA cloning and sequencing we determined the DNA methylation pattern in genomic regions designated site A (which includes DMR- 2) and two CpG islands at sites B and C (Figure 1A). DNA methylation analysis indicated that in normal bone tissues, the imprinting pattern (~50% DNA methylation level with one allele densely methylated and another free of methylation) was identified at site A (Figure 1B). However in osteosarcoma SaOS2 cells, an imprinting defect of hypermethylation was found at site A compared to normal bone tissues. CpG island sites B and C were also densely methylated (~90%) at both alleles. DNA methylation analysis confirmed the locations of these DMRs in normal bone tissues and SaOS2 cells.

To determine whether imprinting defects at these DMRs are associated with osteosarcoma, we analyzed the methylation pattern of all three DMRs (DMR-1, –2 and –3) (Figure 1C) in 8 normal bone tissues and 32 osteosarcoma samples (Supplementary Table 1) by bisulfite-pyrosequencing. With the exception of 5 osteosarcoma samples (FT–7, –422, –426, –430 and –436), all samples were collected during local control surgeries prior to neoadjuvant chemotherapy and/or radiation. Based on our sample age distribution [31], we defined the early- and late-onset OS. 23 samples were early-onset OS (< 30 years) and 9 samples were late-onset OS (> 30 years). Our DNA methylation analysis revealed that imprinting defects, specifically hypomethylation, at DMRs were detected in most of the early-onset osteosarcoma patient samples but were not present in the late-onset samples (Figure 1D). DMR-2 appeared to be most prone to imprinting defects.

### 14q32 index identifies normal/early- and late- onset osteosarcoma

**Figure 2:**
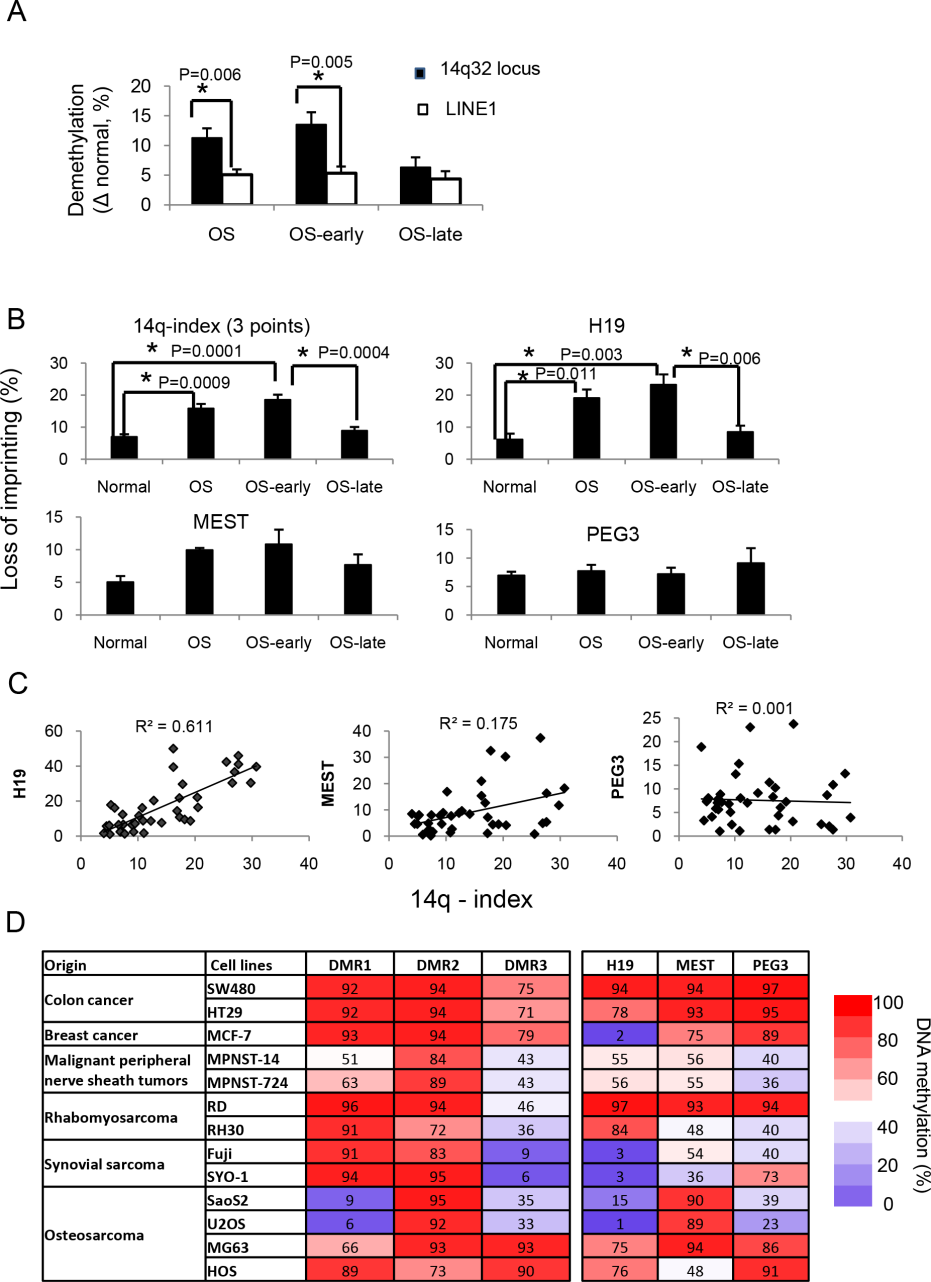
Imprinting defects at the 14q32 locus in osteosarcoma (**A**) Significant differences in demethylation levels between DMRs at 14q32-locus and LINE1 in osteosarcomas. (**B**) Determination of imprinting defects at 14q32 DMRs, H19, MEST and PEG3 in normal bone tissue (normal), osteosarcoma samples (osteosarcoma), osteosarcoma early-onset (osteosarcoma-early) and late-onset (osteosarcoma-late) samples. The imprinting defect extent is defined as the absolute methylation difference between patient samples and the proposed normal imprinted DMR levels (50% methylation). (**C**) Correlations between 14q-index and imprinting defects in H19, MEST, and PEG3, respectively. (**D**) DNA methylation patterns of 3 DMRs at 14q32-locus and 3 other imprinted loci in cancer cell lines representing 6 cancer types. *P < 0.05. Abbreviations used: OS: total OS samples, including both early- and late- onset samples; OS-early: early-onset OS samples (*n* = 23); OS-late: late-onset OS samples (*n* = 9).

To test whether imprinting defects at the 14q32 locus were the consequence of global hypomethylation, we assessed the genome-wide methylation status in osteosarcomas by measuring the methylation of LINE-1, representing 17% human genomic DNA [32] (Figures 1E and 2A). We found that the methylation status of a single allele of LINE-1 decreased by ~5% in both early- and late-onset osteosarcoma. However, the methylation decrease at 14q32-DMRs was ~19% in early-onset osteosarcoma and was significantly higher than that of LINE-1 (*P* = 0.0046). Hypomethylation at the 14q32-locus was therefore not entirely the result of genome-wide demethylation in early-onset osteosarcoma. In contrast, the hypomethylation at 14q32 in late-onset osteosarcoma samples was ~6%, similar to that of LINE-1, suggesting that demethylation in late-onset osteosarcoma was likely due to global hypomethylation.

A pattern of either hyper- or hypomethylation at DMRs signifies imprinting defects. Although hypomethylation was the predominant pattern at DMR- 2 in the majority of cancers compared to normal bone tissues, a few cases did present with hypermethylation at 14q32-DMRs. Thus, we calculated the extent of imprinting defects as the absolute difference of DNA methylation deviation from the normal imprinted-DMR levels (50%). Both gain and loss of methylation at DMR were defined as loss-of-imprinting and both types of changes led to similar gene expression patterns, therefore we used the absolute value of the difference between samples and the theoretical normal (50%) to define the extent. We termed this quantitative deviation of imprinting the *‘14q-index’ (14q-I)*. The 14q-index was calculated as the average extent of imprinting defects at all three DMRs using the following formula:

*14q-index = (│DNAmethy*_DMR−1_ −*50│+│DNAmethy*_DMR−2_ −*50│+│DNAmethy*_DMR−3_ −*50│)/3*

Based on the above equation, the calculated *14q-index* for normal bone tissues was 7.0. It was notable that normal/early-onset osteosarcoma (12–30 years) samples showed a significantly higher *14q-index* at 18.6. The average *14q-index* was 8.5 for late-onset osteosarcoma (> 30 years), a non-significant change versus normal bone tissues (Figure 2B). Based on a 95% confidence interval (CI), we defined imprinting defect as a 14q-index values > 8.5. Twenty of the 23 (87%) early-onset osteosarcomas were 14q-I(+). However, in the late-onset group only 5 of 9 (56%) were positive and the degree of 14q-index changes was not as dramatic as that of the early-onset group.

### Loss of 14q32-locus imprinting is unique to osteosarcoma

Next, we investigated whether the imprinting defect was limited to DMRs in the 14q32-locus or extended to others. We tested the DNA methylation levels of three other known DMRs at the *H19*, *MEST* and *PEG3* imprinted gene clusters in normal bone and osteosarcoma samples. Based on a 95% confidence interval of normal bone tissue, ~59% (19 out 32) of osteosarcoma samples showed imprinting defects at H19-DMRs (Figure 2B). The correlation between imprinting defects at 14q32-DMRs and H19-DMR was 0.78 (R^2^ = 0.61), potentially implicating both loci in tumor development (Figures 1E, 2C). The methylation patterns of H19-DMRs, were much more complex than that of the 14q32-locus, which had both gain- and loss-of DNA methylation (Figure 1E) and the frequency of loss-of-imprinting at H19 was much lower than that at 14q32-locus. Imprinting defects were not found in DMRs of *MEST* and *PEG3* and there was no correlation between either of these two genes and the 14q32-locus (Figure 2B and 2C).

We then tested whether imprinting defects at the 14q32-locus were unique to osteosarcoma or widespread among different cancer types. The DNA methylation levels of 14q32-, H19-, MEST- and PEG3-DMRs were determined using 13 cell lines representing 6 different cancer types (Figure 2D). Hypomethylation at 14q32 IG-DMR (DMR- 1), which was characteristic of human osteosarcoma tissue samples, was also observed in osteosarcoma cell lines SaOS2 and U2OS. However hypermethylation patterns were observed in osteosarcoma cell lines MG63 and HOS both characterized as having an osteoblastic phenotype. There were no consistent patterns of change in DNA methylation status in the 6 additional cancer types tested, indicating that hypomethylation of this set of DMRs may be a distinguishing feature of osteosarcoma.

### Downregulation of 14q32 locus genes is associated with imprinting defects

**Figure 3:**
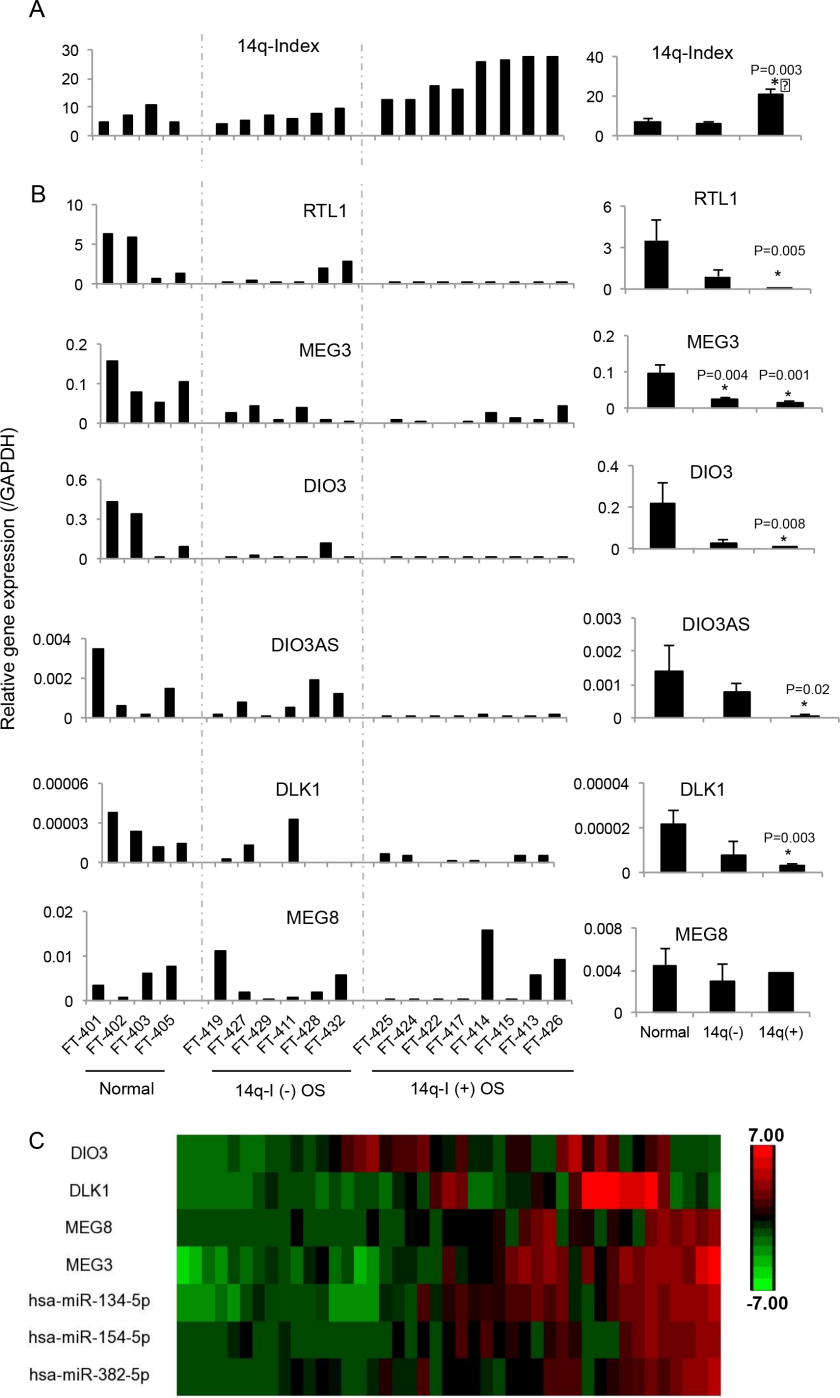
Downregulation of genes by imprinting defects at the 14q32-locus (**A**) 14q index (14q-I) of four normal bone tissue samples, six 14q-I(−) osteosarcoma and eight 14q-I(+) osteosarcoma samples. (**B**) mRNA levels of six imprinted genes by qRT-PCR with GAPDH used as control. The right column indicates the average and standard error of the imprinted gene expression levels in 3 different groups, including normal bone tissues, 14q-I(−) osteosarcoma and 14q-I(+) osteosarcoma groups, respectively. *P < 0.05. (**C**) Relative expression levels of representative DLK1-DIO3 cluster mRNAs and 14q32 miRNAs from an independent cohort of 43 osteosarcoma patient samples.

Imprinting defects at DMRs have been shown to regulate the expression of genes within the locus. To determine the effects of imprinting defects at 14q32-DMRs on gene expression, we measured the expression levels by qRT-PCR of 6 imprinted genes in this locus including paternal (*DLK1, RTL1*, and *DIO3*) and maternal (*MEG3, MEG8*, and *DIOAS*) genes expressed in 4 normal bone tissue samples, six 14q-I(−) and eight 14q-I(+) osteosarcoma samples (Figure 3). *RTL1*, *DIO3*, and *MEG3* were relatively highly expressed in normal bone tissues, while *DLK1*, *MEG8* and *DIO3AS* were expressed at lower levels. Most of the imprinted genes at the 14q32-locus (with the exception of MEG8) were downregulated in 14q- I(−) samples, and further decreased in 14q-I(+) samples.

To evaluate the co-expression of imprinted genes and miRNAs at the 14q32 locus, we analyzed the expression of DLK1-DIO3 cluster genes (*DIO3*, *DLK1*, *MEG3*, and *MEG8*) and representative 14q32 miRNAs (miR-134, miR-154, and miR-382) in an independent set of 43 osteosarcoma tumor samples. In the majority of tissues analyzed, 14q32 miRNA expression was correlated with DLK1-DIO3 cluster genes, particularly with *MEG3* and *MEG8* (Figure 3C).

### Histone modifications at the 14q32 imprinted region

**Figure 4:**
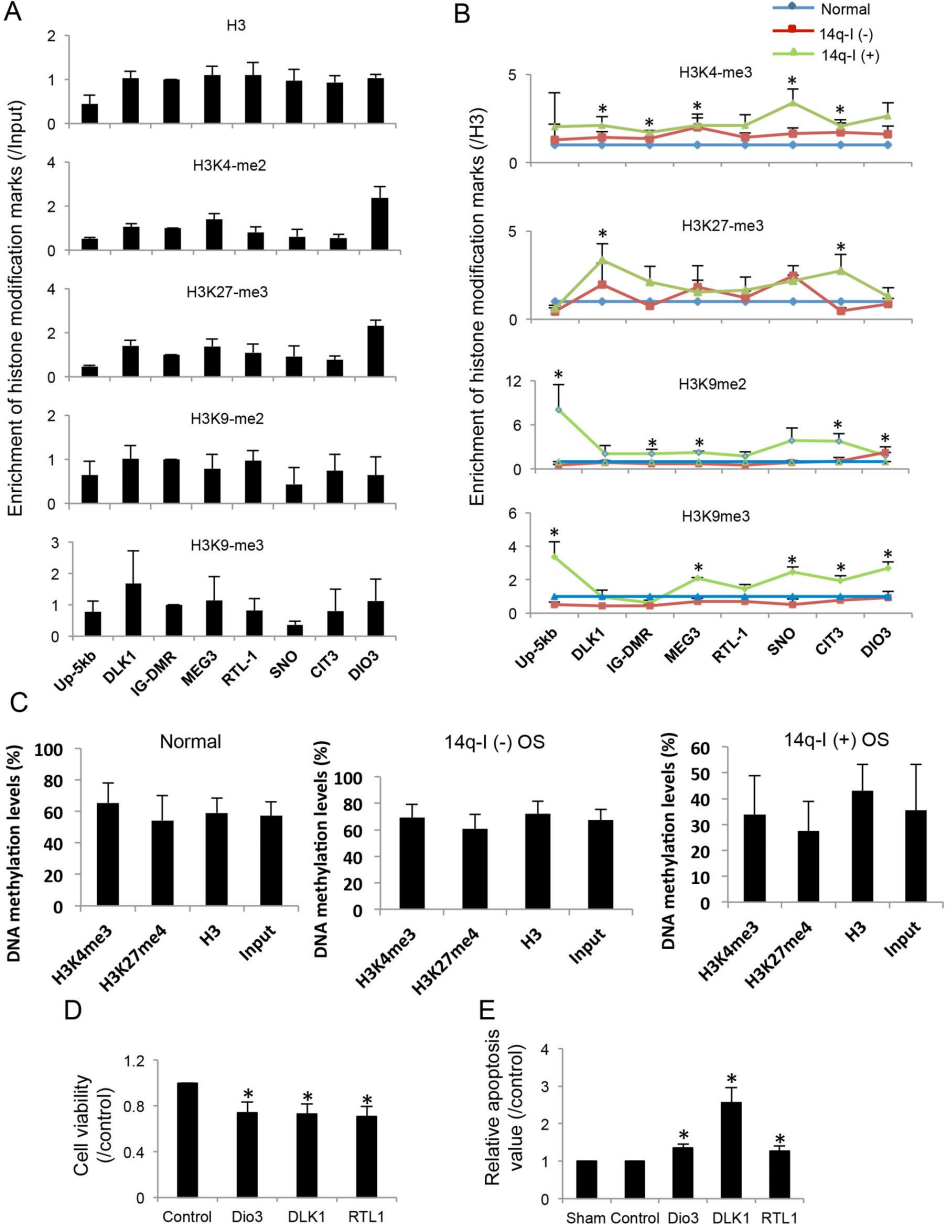
Histone modifications around imprinted genes and their potential tumor suppressor functions at the 14q32-locus (**A**) Enrichment of H3, H3K4-me3, H3K27-me3, H3K9-me2 and H3K9-me3 at the promoter regions of imprinted genes in normal bone tissues. Input was used as internal control. Up-5kb: site ~5 kb upstream of the promoter of DLK1; SNO: small non coding RNA region; CIT: miRNA cluster 3 region. (**B**) H3K4-me3, H3K27-me3, H3K9-me2 and H3K9-me3 enrichment normal bone tissues, 14q-I(−) and 14q-I(+) osteosarcoma samples. H3 was used as internal control. *P < 0.05. (**C**) DNA methylation levels of the ChIP samples pulled down by H3, H3K4-me3, and H3K27-me3 antibodies in normal bone tissues, 14q-I(−) osteosarcoma and 14q-I(+) osteosarcoma samples. Input sample was used as control. (**D**) Significant decrease of cell viability by overexpression of DIO3, DLK1 and RTL1 in SaOS2 cells. Paired *t*-test was performed. *P < 0.05. (**E**) Increased apoptosis by overexpression of DIO3, DLK1 and RTL1 in SaOS2 cells. pCDNA3.1 empty vector was used as control. Sham represents SaOS2 cells without plasmid transfection. Results represent the average of three independent transfections. *P < 0.05.

Histone modifications regulate long distance gene expression. We measured histone modification patterns at the 14q32-locus with ChIP assays by mapping the active marker H3K4-me3 and the silence markers H3K27- me3, H3K9-me2 and H3K9-me3 in normal bone tissues, 14q- I(−) and 14q-I(+) osteosarcoma samples. Among the normal samples, we found that the imprinted gene cluster from *DLK1* to *DIO3* shares a similar histone modification pattern, including H3, H3K4-me3, H3K27-me3, H3K9-me2 and H3K9-me3 (Figure 4A). The 14q32-locus was enriched with both active marker H3K4-me3 and silence markers H3K27-me3, H3K9-me2, and H3K9-me3 in normal bone tissues (Figure 4A). GAPDH, cMYC, p16, and RARB were used as controls; and IgG was used as a negative antibody control to evaluate the enrichment of histone modification markers [33] (Supplementary Figure 1A–1D).

Because the entire imprinted region shares a similar histone modification pattern, we used levels of different histone markers at IG-DMR to represent the histone modification at this genomic region. H3K4-me3 at 14q32 IG-DMR was lower than *GAPDH*, but was ~80-fold higher than the *IgG* negative control. On the other hand, H3K27-me3 at 14q32 IG-DMR was 1.6-fold higher than at *RARB* and was ~180-fold higher than IgG negative control (Supplementary Figure 1E–1F). Similarly, H3K9- me2 and H3K9-me3 were detected at much higher levels at IG- DMR than IgG. Based on these observations we concluded that H3K4-me3, H3K27-me3, H3K9-me2, and H3K9-me3 were enriched at the 14q32-imprinted region.

We examined the changes to histone modification in osteosarcoma and looked for differences between 14q-I(−) and 14q-I(+). We found the total H3 levels were decreased in both 14q-I(−) and 14q-I(+) samples (Supplementary Figure 2A). In 14q-I(+) samples, H3K4- me3, H3K9- me2, and H3K9-me3 were increased, and H3K27-me3 was decreased. But in 14q-I(−) samples, all of the histone markers showed a decrease. After normalizing to the level of H3, the majority of histone markers were increased in 14q-I(+) osteosarcoma group (Figure 4B and Supplementary Figure 2), but were decreased or unchanged at most of imprinted gene loci in 14q-I(−) osteosarcoma. We deduced that all of the imprinted genes were regulated by histone modification as a single unit and silenced by the increase of three silencing markers in the 14q-I(+) region.

To confirm that the sonication process did not preferentially disrupt either allele, we used total input as a control. In all three groups, the DNA methylation levels of the input samples were consistent with the results we obtained directly from patient DNA, suggesting that sample preparation during the ChIP assay did not affect the measurements.

DNA methylation levels for both H3K4-me3 and H3K27-me3 ChIP samples shared a similar pattern with the input control in all samples (Figure 4C), confirming that the H3K4-me3 and H3K27-me3 were equally present at both maternal and paternal alleles.

### Proteins encoded by the 14q32 locus act as tumor suppressors

It has previously been reported that *MEG3* is a tumor suppressor gene [15], but *RTL1* and *DIO3* remain candidate genes of interest and have not been well studied in osteosarcoma. Transient overexpression of *DLK1*, *RTL1* or *DIO3* individually in SaOS2 cells decreased cell viability by ~30%, compared to cells transfected with control plasmids (Figure 4D). Individual transfection of *DLK1*, *RTL1* or *DIO3* also significantly increased apoptosis in SaOS2 cells (Figure 4E). Our previously published work has demonstrated that several miRNAs in this area can also function as tumor suppressors in the context of osteosarcoma [11].

### Imprinting instability at 14q32 locus

It is likely that osteosarcoma patients inherit, at least in part, imprinting instability from their parents, which predisposes them to the early-onset oncogenesis typical of osteosarcoma. To test this hypothesis, we measured DNA methylation levels of two IG-DMRs at the 14q32 locus in buccal samples from 12 unrelated normal subjects (median age 26 years), and 10 sets of triad samples (10 osteosarcoma patients plus their biological parents). Due to the rapid turnover of the buccal mucosa, epithelial cells are subject to an increased risk, compared to normal bone tissue, of changes in DNA methylation status and of development of loss-of-imprinting resulting from age- related methylation mechanisms [34]. In unrelated normal individuals, we found the average methylation level of DMR-1 was 53% and DMR-2 was 64%, likely due to normal age-related increases in DNA methylation. The normal range of DNA methylation in buccal tissues, was therefore defined as 51–55% at DMR-1 and 61–67% at DMR-2 (using a 95% CI) (Supplementary Table 3A).

**Figure 5:**
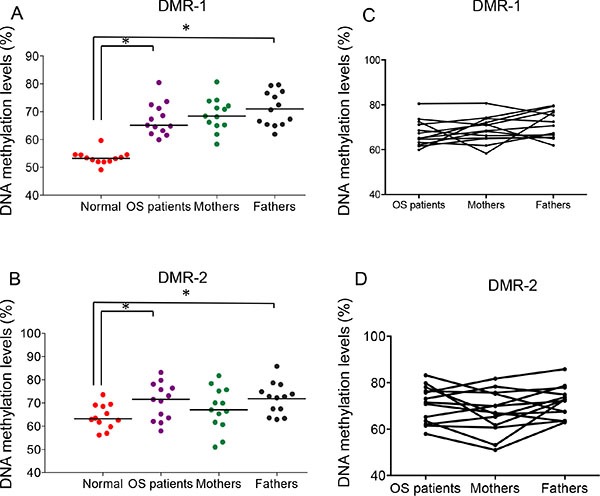
Imprinting defects at 14q32-locus in buccal tissue of osteosarcoma patients and their biological parents (**A**) and (**B**) DNA methylation levels of DMR-1 and DMR-2 of buccal samples from normal subjects, osteosarcoma patients and their parents. One-way ANOVA was performed. *P < 0.05. (**C**) and (**D**) The overlay graph of the DNA methylation levels of DMR-1 and DMR-2 of the OS family buccal samples.

Hypermethylation imprinting defects at IG-DMRs, especially DMR-1, were detected in all of the analyzed osteosarcoma patient samples and, importantly, in all parent samples. There was a significant difference in DMR-1 (*P* < 0.0001) and DMR-2 (*P* < 0.05) DNA methylation levels between unrelated normal healthy samples and those in the osteosarcoma patient or parent samples (Figure 5A, 5B and Supplementary Table 3B). Patient DMR methylation patterns appear to be correlated with their respective parents' patterns (Figure 5C–5D). The Pearson's correlation coefficient (Pearson's r) is 0.5 between osteosarcoma patients and their fathers at DMR-1, and is 0.63 between osteosarcoma patients and their mothers at DMR-2 (Supplementary Table 3C). This suggests that the imprinting defect at 14q32 might be inherited from existing parental imprinting instability, especially from the paternal side.

### Imprinting defects did not result from pre- existing mutations in spontaneous osteosarcoma mouse models

To determine if imprinting defects at the 14q32 locus were a result of pre-existing mutations during osteosarcoma development, we studied tumor tissues using a spontaneous osteosarcoma model in *T2/onc*, *p53*^R270 H/+^, *OSx-Cre, ROSA26-LSL-SB11* mice. These mice express a *p53*^R270 H^ point mutation and allow random insertional mutagenesis using the *Sleeping Beauty* (*SB*) transposon system. Consistent with a previous report [35], ~60% of *p53*^R270 H/+^ mice developed osteosarcoma with an average age of onset of ~16 months. In *T2/onc*; *p53*^R270 H/+^*;OSx-Cre; ROSA26-LSL-transposase* mice, 70% of mice developed osteosarcoma by ~11 months with the earliest case appearing at 8 months. *SB* mutagenesis led to accelerated tumor formation compared to the *p53*^R270 H/+^ mice.

**Figure 6:**
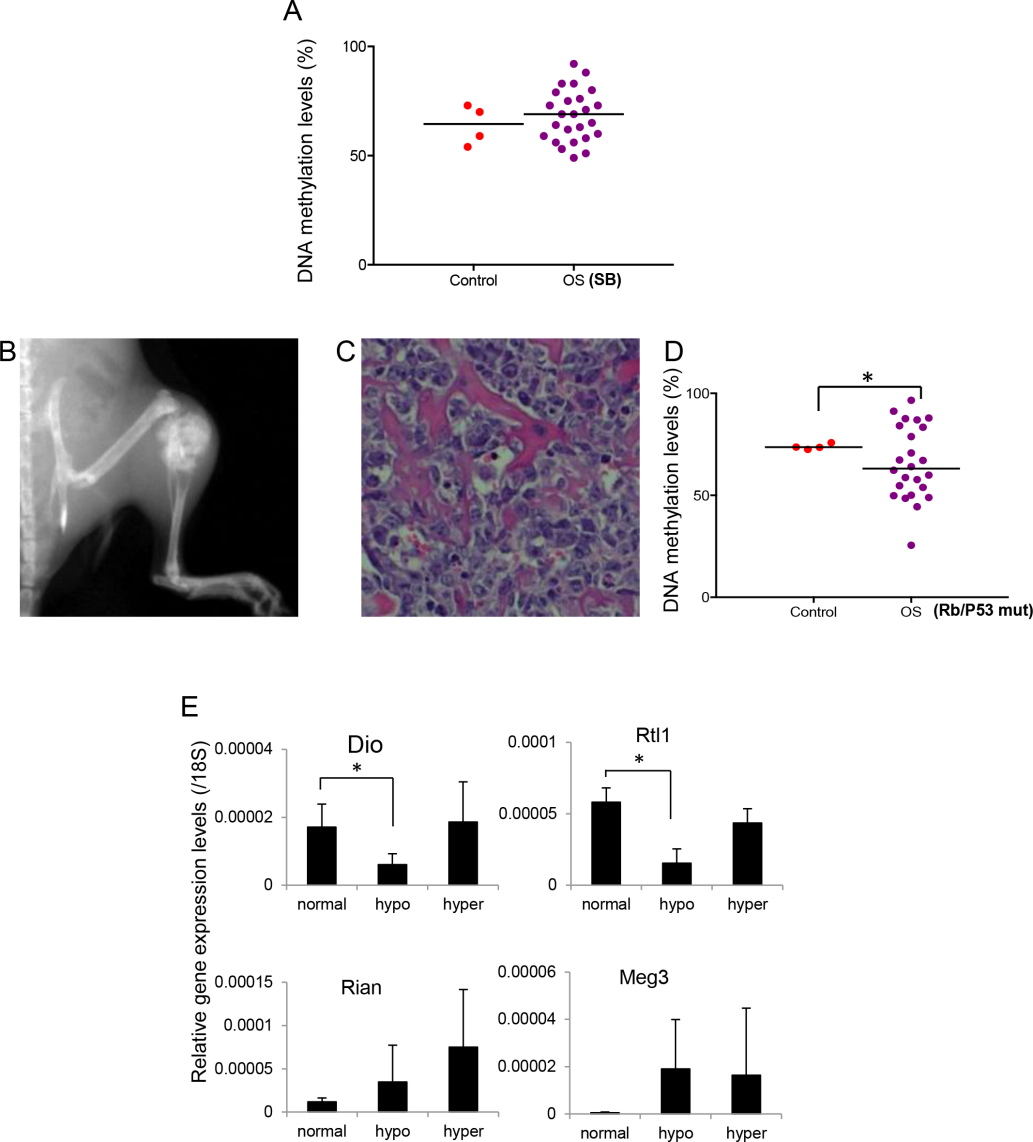
DNA methylation changes at 12qF DMR of spontaneous OS mouse models (**A**) DNA methylation levels of 12 qF DMR of Sleeping Beauty (T2/onc; p53R270H/+;OSx-Cre; ROSA26-LSL-SB11) induced OS samples. (**B–C**) Representative radiograph and histology section of osteosarcoma in mice homozygous for conditionally disrupted alleles of Trp53 and Rb1. (**D**) DNA methylation levels of 12qF DMR of p53/Rb1mut induced OS samples. *P < 0.05. (**E**) Expression levels of imprinted genes in 12 qF region in the p53/Rb1mut induced OS samples. 18 s rRNA was used as internal control. *P < 0.05.

We determined the DNA methylation changes of IG-DMR at the mouse 12qF locus (homologous to human 14q32), with bone tissues from normal mice as controls. In normal bone samples, the mean DNA methylation level was 64% (95% CI: 55% – 73%) (Figure 6A and Supplementary Table 4A). In Trp53-R270 H/SB tumor samples, 44% (11 out of 25) showed imprinting defects, which was comparable to the prevalence of imprinting defects in late-onset human osteosarcoma (~56%), suggesting that late-onset osteosarcoma in humans is more likely to be caused by spontaneous somatic mutations. In contrast to human osteosarcoma samples, imprinting defects in these mice were due to hypomethylation (3 out of 25) or hypermethylation (8 out of 25). As reported earlier, it is likely that different regulatory mechanisms are involved in human and mouse at this locus [29]. In humans, DMRs are located outside CpG islands, while in mice the DMRs are located within CpG islands, which are better protected against methylation changes. The fact that 12qF imprinting defects were found in only 44% of *SB*-induced osteosarcoma suggested that 12qF imprinting defects were not caused by osteosarcoma formation, but might instead be one of the underlying mechanisms predisposing the tumor's development.

### Imprinting defects at 12qF were related to osteosarcoma development in p53/Rb mutation mouse model

To further establish the role of imprinting defects at 12qF in osteosarcoma development, we generated an osteosarcoma mouse model with both p53 and Rb mutations (Figure 6B–6C). 25 mice with osteosarcoma representing various tumor locations (20 limb, 3 pelvis, 1 neck, and 1 soft tumor inside the chest wall) were included in the study. We were able to determine the DNA methylation levels at the 12qF IG-DMR locus in 24 of these samples. Based on 95% CI of normal bone tissue, we defined that normal methylation range was 72~76% (Supplementary Table 5). Imprinting defects were found in all osteosarcoma samples, consistent with observations in human osteosarcoma (87%). We found both hypomethylation (66.7%; 16 out of 24) and hypermethylation (33.3%; 8 out of 24), similar to the previous mouse osteosarcoma samples (Figure 6D).

We examined the expression levels of the homologous 14q32 locus imprinted genes in these mouse osteosarcoma samples. We found that *Dio3* and *Rtl1*, the paternally expressed genes, were dramatically decreased in the DMR hypomethylated samples but had no significant change in the hypermethylated samples (Figure 6E), again consistent with the human osteosarcoma samples (Figure 3). The maternally expressed genes *Meg3* and *Rian* had no significant changes in expression although an increased trend was observed (Figure 6E), in contrast to the human samples which saw little overall change in expression.

### 14q-index is a prognostic indicator in osteosarcoma

**Figure 7:**
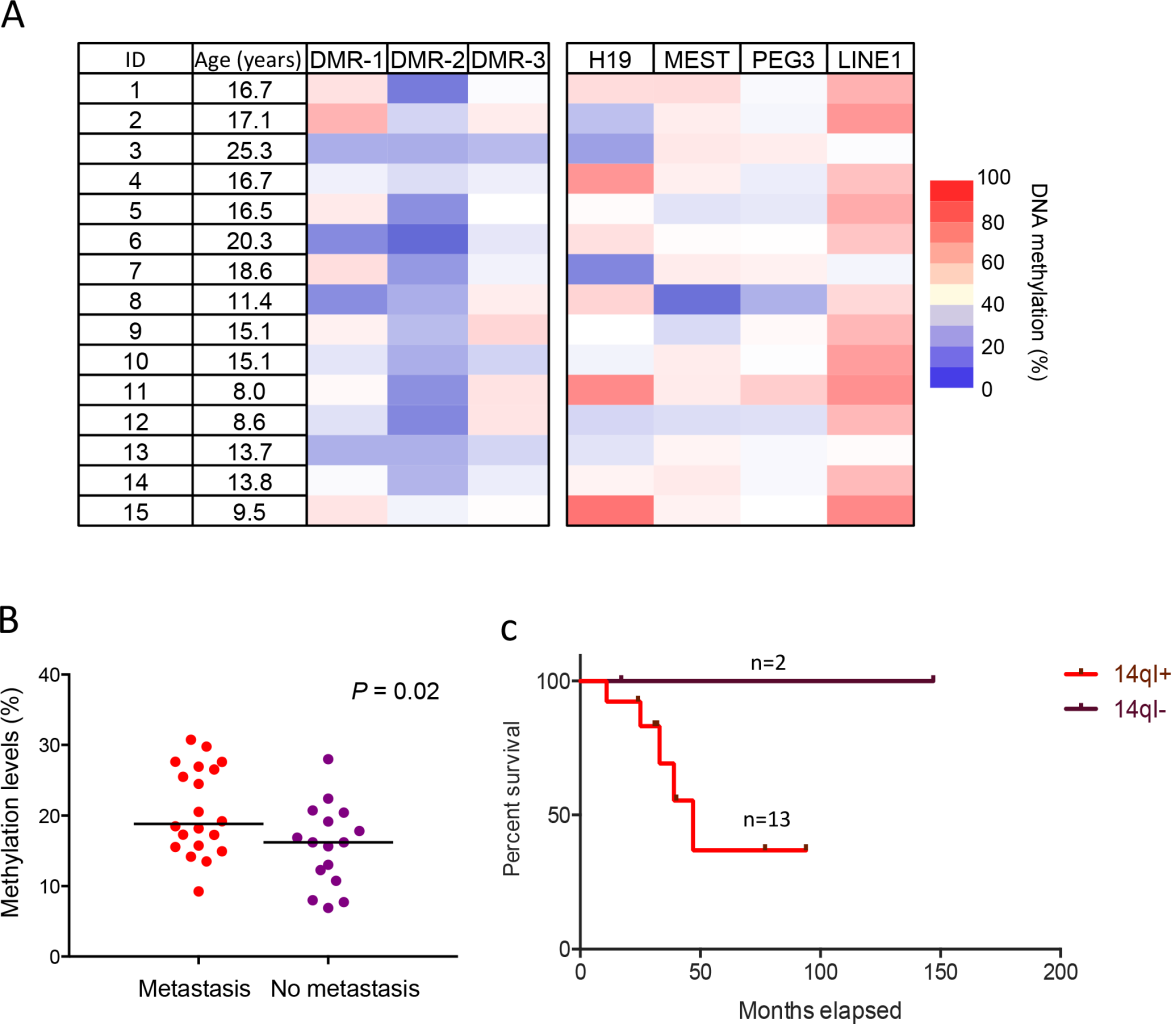
14q-index as prognostic indicator in osteosarcoma (**A**) DNA methylation levels of DMRs of 14q32-locus, three other imprinted loci, including H19, MEST and PEG3, and methylation levels of LINE1 in 15 biopsy samples analyzed by bisulfite pyrosequencing. (**B**) The difference of 14q-index between patients with and without metastasis in early-onset OS. (**C**) Survival analysis of OS patients with different 14q-index.

To determine whether the 14q-index can serve as a prognostic indicator in osteosarcoma, we tested an independent cohort of 15 human osteosarcoma biopsy samples obtained prior to chemotherapy/radiation with long-term clinical follow-up data. We again found significant imprinting defects at multiple loci, especially DMR-2 (Figure 7A), validating our previous results. Hypomethylation defects, especially at DMR-2, were present in the majority of samples and 86.7% (13 out of 15) samples showed an increase in the 14q-index. Combining these 15 new samples with the initial 21 osteosarcoma samples (onset at < 30 years), we found a significant difference in the 14q-index in osteosarcoma patient samples with and without metastasis (Figure 7B). Additionally, osteosarcoma patients with imprinting defects and a high 14-q index had poorer survival outcomes (Figure 7C). With this limited sample size, a robust statistical analysis was not possible, however it was suggestive that patients with a normal 14q-index do have a survival advantage.

## DISCUSSION

The results of our study suggest that hypomethylation at 14q32 - imprinted IG-DMRs is predominantly associated with osteosarcoma (onset < 30 years) and is a likely mechanism of downregulation of imprinted genes and miRNAs at this locus. Although hypomethylation typically leads to increased gene expression, it has been reported that hierarchical interaction of the methylation pattern in the three DMRs at the 14q32 locus does have a negative effect on gene expression [36, 37]. More importantly, the observed imprinting defects in 14q32 DMRs did not appear to be due to genome-wide methylation changes. DNA methylation patterns of 14q32-DMRs in osteosarcoma appear to be significantly different than those observed in other cancers and may provide additional insights into the pathobiology of osteosarcoma. We developed the ‘14q-index’ (14q-I) to quantitate the degree of imprinting defects in osteosarcoma and propose that 14q-I(+) can be used as both a predictive marker of osteosarcoma and a prognostic indicator as patients with a normal index value tend to have improved survival outcomes.

We studied the effects of 14q32-locus imprinting defects on the expression of genes at this region. Notably, all the genes and miRNAs tested showed different degrees of downregulation [11]. Our data demonstrating that 14q32 locus genes (with the exception of *MEG8*) are significantly downregulated in osteosarcoma samples contradicts a previous report that loss-of-imprinting causes differential changes in paternal and maternal expressed genes and an inverse correlation at the IGF2-H19 locus [38]. This may be due to different regulatory mechanisms at the 14q32 and H19-IGF2 loci [39]. The imprinted genes at the 14q32-locus can be co-expressed in mesoderm-derived tissues and inversely expressed in lung, liver, and placenta [40]. It is likely that both the maternal and paternal imprinted genes are regulated as a single unit in bone tissue and imprinting defects can cause co- repression of both maternal and paternal expressed genes in osteosarcoma. It is interesting to note that imprinting defects have a differential impact on gene expression. In mice, the paternally and maternally expressed genes were inversely regulated with imprinting defects. However, in humans these genes were affected uniformly regardless of their parental origin, which may be due to the observed histone modifications.

There is a correlated pattern of 14q32 miRNA and *DLK1-DIO3* gene cluster expression within individual patient samples. The genes at both ends of the cluster, *DLK1* and *DIO3*, showed more variability and this may implicate additional regulatory factors. Previously, we reported that significant downregulation of miRNAs from this locus was correlated with poor prognosis in human and canine osteosarcoma [41]. Consistent with these observations, our study also revealed that *DLK1*, *RTL1*, *DIO3,* and miRNAs in this region are candidate pro-apoptotic genes [20]. MEG3 has been previously reported to function as a tumor suppressor that can regulate p53 expression [15]. Our results also suggested that imprinting defects at *MEG3*-DMR (DMR-3) were not a significant contributor to cancer development but could potentially hasten the disease process. This finding was consistent with previous results suggesting that IG-DMRs are germline-derived primary DMRs, while *MEG3*-DMR is a post-fertilization-derived secondary DMR [42]. The IG-DMR acts hierarchically to regulate the methylation status of the somatic *Glt2*-DMR (homolog to human *MEG3*- DMR) in mice. Previously, we have reported that several 14q32 miRNAs function as tumor suppressors by targeting cMYC and that blocking these miRNAs using antimiRs in normal human osteoblasts was transformative [11]. Taken together, these data suggest that the 14q32 locus contains multiple tumor suppressors.

It has previously been suggested that the 14q32-imprinted locus may share similar regulatory mechanism(s) such as the IGF2-H19 imprinting cluster [43]. However, the patterns of co-expression or co- silencing are only found in the 14q32-locus, which implies different regulatory mechanisms between the two imprinted regions. We found that both the active marker H3K4-me3 and silenced marker H3K27-me3 were enriched in this 14q32-locus. We speculate that this locus is potentially controlled as a ‘bivalent domain,’ which is also associated with developmental and differentiation functions [44]. A bivalent domain is defined as the presence of both H3K4-me3 and H3K27-me3 at the same locus on the same allele simultaneously. We used the 14q32 IG-DMR region's DNA methylation status to define each allele's parental origin. The maternal allele is characteristically free from methylation while the paternal allele is densely methylated. Although Single Nucleotide Polymorphism (SNP) analysis would further confirm the presence of bivalent domain, we did not find suitable SNPs in this region for testing. These results suggested a possible mechanism for silencing of the entire 14q32-imprinted genes and miRNAs in osteosarcoma samples, especially in 14q-I(+) osteosarcoma cases. We also found that this entire imprinted region might be regulated as a single unit in bone tissues with the boundary marked between *DLK1* and *DIO3*.

Imprinting defects at the 14q32-locus were found in 87% of osteosarcoma samples (onset < 30 years). The specific loss of imprinting at the 14q32 and H19 loci in osteosarcoma samples suggested that these methylation changes were disease-specific and not the result of global methylation changes. Comparison of the DNA methylation patterns of 14q32 IG-DMRs in the buccal DNA samples of osteosarcoma patients, their biological parents, and unrelated normal subjects implied that imprinting instability exists in the affected child's parents but is absent in unrelated normal subjects. We speculate that inherited imprinting defects at the 14q32 locus may contribute to pediatric/young-adult development of osteosarcoma. Given the apparently ‘normal’ phenotype of the parents, imprinting instability itself is likely insufficient to cause cancer, but might predispose affected subjects to osteosarcoma.

It is of interest to note that osteosarcoma occurs predominantly during puberty, a period characterized by accelerated linear bone growth, and some imprinted genes in these regions have been implicated in bone differentiation. It is likely that under growth stimulation, patients with imprinting defects at 14q32 and associated silencing of multiple tumor suppressor genes are at greater risk of developing osteosarcoma. Similarly, loss-of-imprinting at this locus is implicated in other cancers such as lymphoma, colon cancer, and neuroblastoma [45], which is also supported by our analysis of the imprinting status at 14q32-DMRs of 10 cancer cell lines representing six cancer types. In our study we clearly identified the differential methylation patterns in the normal (< 30 years) and late-onset osteosarcoma groups. It is possible that some of the pre-operative treatments that these patients received might also affect the epigenetic patterns although our sample collection criteria attempted to minimize this possibility. The timing of buccal sampling in patients with osteosarcoma may also have affected results, as some osteosarcoma drugs cause aggressive mouth sores [46].

Several mouse uni-parental disomy (UPD) models (pUPD12 and mUPD12) have been studied previously; these models are non-viable with skeletal and skeletal muscle defects. They suggested that imprinted genes on chromosome 12 are essential for the development of the mesodermal and neural crest-derived lineages that give rise to bone [47]. Skeletal abnormalities such as small hands, hyper-extensible joints and advanced bone age have been reported in human mUPD14 patients [48]. In osteosarcoma, imprinting defects at the 14q32-locus can cause significant downregulation of imprinted genes and miRNAs, which may further contribute to its pathobiology and disrupt normal skeletal development.

We generated mouse models of osteosarcoma by disrupting *Trp53* in combination with either *SB* transposon-mediated mutagenesis or concomitant loss of *Rb1*. We found that imprinting defects at 12qF, the homolog to the human 14q32 locus, were quite uncommon in *SB*-induced osteosarcoma. In fact, their frequency was similar to that of human late-onset osteosarcoma, which is consistent with the hypothesis that mutations play a more causative role in the late-onset osteosarcoma development. In contrast, all *Rb1/Trp53*-mutant osteosarcoma mice had imprinting defects at 12qF DMRs, confirming that 12qF-imprinting defects are an important mechanism for osteosarcoma development in this model.

Similar to our observations in normal/early (< 30 years) and late-onset human osteosarcoma, there were distinctive 12qF locus methylation patterns between the two mouse models that may, in part, correspond to their genetic differences. The high frequency of imprinting defects in both the *Rb1/Trp53* model and in human osteosarcoma, suggested the presence of one or more conserved mechanisms in osteosarcoma development. *Rb1* and/or *Trp53* mutations along with imprinting defects may cooperatively function towards development and progression of osteosarcoma. The *SB* mouse osteosarcomas have less genomic instability and chromothripsis, compared with *Trp53* (no *SB*) or *Rb1/Trp53* mouse osteosarcomas [49]. Further, only about 20% of *SB* mice showed common insertion site mutagenesis in *Rb1* [50]. *SB*-induced tumors may represent a model for the pathogenesis of late-onset osteosarcomas, which typically have had radiation exposure, prior alkylating chemotherapy, Paget's disease, or other mutation-generating conditions or exposures. With rampant *SB* mutations driving oncogenesis, epigenetic alterations may not be required to further drive the pathogenesis. Without a *SB* mutagenesis program, it is possible that osteosarcoma development may require epigenetic modifications to induce cancer growth and maintenance. RB1 has epigenetic functions, which have not been well sorted out in osteosarcoma but previous publications have reported on the significant role of RB-loss in osteosarcoma [51, 52].

The overall change in 14q32 - DMR methylation status, quantitatively described as the 14q-index, presents a promising indictor of diagnostic and prognostic significance that will need to be validated in a larger patient set. Using osteosarcoma patient samples and two mouse osteosarcoma models, our results indicate that normal/early-onset osteosarcoma (onset < 30 years) is characterized by inherited imprinting defects that may contribute to osteosarcoma pathobiology, in contrast with late-onset osteosarcomas that appear to be driven more by different factors. These findings may provide new opportunities for improving the diagnosis and treatment options for osteosarcoma patients.

## MATERIALS AND METHODS

### Cell lines, patients and normal tissue samples

FUJI, SYO-1, HT-29, SW480, MPNST-14, MPNST-724, RD, RH30, MCF-7, SaOS2, U2OS, MG63, and HOS cell lines were used for methylation analyses, and cultured under established conditions [11, 53]. Colon cancer cell lines HT-29 and SW480, synovial sarcoma cell lines SYO-1 and FUJI, rhabdomyosarcoma (RMS) cell lines Rh30 and RD, MPNST cell lines MPNST-14 and MPNST-724, breast cancer cell line MCF7, and osteosarcoma cell line SaOS2 were used in this study. Synovial sarcoma cell lines FUJI and SYO-1 were obtained from Dr. Torsten Nielsen (University of British Columbia), colon cancer cell lines HT-29, SW480, and MCF7 were kindly provided by Drs. Clifford Steer and Kathryn Schwertfeger (University of Minnesota), RMS cell line Rh30 was obtained from ATCC (CRL-2061), and RMS cell line RD was provided by Dr. Peter Houghton (St. Jude's Children's Research Hospital). MPNST cell lines were generously provided by Dr. Jonthan Fletcher, Harvard Medical School.

Osteosarcoma tissue samples were obtained from the tissue procurement facility at the University of Minnesota or from the Cooperative human tissue network. Dr. Logan Spector provided triad buccal DNA samples. All patient samples were obtained according to the University of Minnesota institutional review board (IRB) approved protocols. Osteosarcoma tissue samples with clinical follow-up were obtained from Leiden University Medical Center, The Netherlands. Available clinical and follow-up information for these samples are provided in Supplementary Table 1.

### DNA and RNA extraction and qRT-PCR

DNA was extracted with a DNA Extraction kit from Qiagen, and RNA extraction was performed with a mirVana Total RNA Isolation kit (Life Technologies). Extractions were carried out according to the manufacturer's instructions. qRT-PCR was performed with 2 μg DNase1 treated total RNA using High Capacity RNA-to-cDNA kit (Applied Biosystems); and PCR amplification was performed with SYBR Green I Master kit (Roche Applied Science) in a LightCycler 480. Primers were listed in Supplementary Table 2. GAPDH and 18 s rRNA were used as internal controls.

### mRNA and miRNA RNA-Seq expression profiling

To perform high-resolution quantification of mRNA and miRNA expression using RNA-Seq, we used at least 1 μg of each pool of total RNA according to the mirVana Total RNA Isolation kit (Life Technologies). We sequenced mRNA libraries using 100 bp paired-end runs on a HiSeq 2000 (Illumina) using v3 chemistry. miRNA libraries were sequenced using 50 bp paired-end runs on a HiSeq 2500 (Illumina) using v4 chemistry. To quality-check raw-sequence FASTQ files, we used FastQC (http://www.bioinformatics.babraham.ac.uk/projects/fastqc/). The TopHat2 algorithm was used to align the mRNA FASTQ files to the Human UCSC hg19 Assembly genome [54]. Next, to quantify the aligned reads against the reference human genome hg19 transcript annotations, we used Cufflinks and Cuffdiff, according to the standard Fragments Per Kilobase of transcript per Million mapped reads (FPKM) methods [55]. To quantitate miRNA expression levels, mature miRNA sequences were used to search miRNA FASTQ files and normalized to Counts per Million total reads for each sample. For statistical analysis and clustering, we used the Partek Genomics Suite software package (Partek Inc). FPKM (mRNA) or Counts per Million total reads (miRNA) values were log base 2 transformed and mean centered before clustering.

### DNA bisulfite pyrosequencing and TA cloning and sequencing

Bisulfate DNA treatment, pyrosequencing and TA cloning and sequencing were performed as previously described [56]. Normal buccal samples were used as control for bisulfite treatment. Two rounds of PCR were carried out to synthesize biotin-labeled specified PCR products with the primers listed in Supplementary Table 2. TA cloning and sequencing were also used in selected samples. Bisulfite-PCR products were cloned into a pCR4.0-TOPO vector and 6~10 clones were selected and sequenced. Methylation levels were calculated as the average value of the selected clones [57].

### Chromatin immunoprecipitation (ChIP) assays

The ChIP assay was performed as previously described [58]. Three ~50 mg tissue samples of different groups (normal bone tissue, 14q index (−), and 14q index (+)) were treated with formaldehyde to cross-link histone to DNA. After sonication and pre-cleaning, the samples were incubated with 3~5 μl anti-H3K4me3, H3K27- me3, and IgG (Millipore) at 4°C overnight, respectively, and then with Dynal Magnetic beads from Invitrogen for 1 hour at 4°C. After washing, immunoprecipitated complexes were eluted and histone-DNA crosslinks were reversed. DNA was extracted, and real-time PCR was used to quantify the immunoprecipitated DNA.

### Plasmid construction, cell proliferation and apoptosis analyses

Human *DIO3* and *RTL1* coding sequences were amplified and cloned into pcDNA3.1 (Invitrogen) by *Xba*I and *Xho*I. Human *DLK1* overexpression plasmid was obtained from Origene. SaOS2 cells were planted in 48 well plates 24 hours before transfection. A total of 0.4 μg overexpression constructs were transfected into SaOS2 cells with lipofectamine. The same amount of pcDNA3.1 empty vector was used as control. Cell proliferation was determined after transfection for 72 hrs using Cell Titer 96 Aqueous One Solution Cell Proliferation Assay (Promega) in a Synergy 2 microplate reader. Apoptosis assays were conducted with the Vybrant apoptosis assay kit #8 (Molecular Probes, Invitrogen) on a BD FACS Canto II according to the manufacturer's instruction.

### Spontaneous osteosarcoma mice models

Tumor and matched normal bone tissue samples obtained from *Sleeping Beauty* [50] (*T2/onc*; *p53*^R270 H/+^*;OSx-Cre; ROSA26-LSL-SB11)* induced osteosarcoma mouse models were used to determine the methylation levels of 12qF DMR regions. Tumor and matched normal bone tissue samples were obtained from mice homozygous for conditionally disrupted alleles of *Trp53* and *Rb1*, induced by *Prx1Cre* as described previously [59]. Schematic representation for generation of (Prx1Cre;Rb1-fl/fl;Trp53-fl/fl) osteosarcoma in mice homozygous for conditionally disrupted alleles of *Trp53* and *Rb1* is given is Supplementary Figure 3. All animal studies were IACUC approved; and details used in the study are provided in Supplementary Tables 4 and 5.

### Statistics

Statistical analysis such as mean, standard deviation, were calculated for each group analyses and the standard error was used as an index for variability. Student two-tailed *t*-test was performed for statistical analysis. The unpaired *t*-test was used to compare patient samples; and the paired *t*-test for apoptosis, cell proliferation assays and buccal tissue of osteosarcoma family member studies. One-way ANOVA was used in the family sample studies. Kaplan-Meier curve was used in the patient survival outcome studies.

## SUPPLEMENTARY MATERIALS TABLES AND FIGURES


