# Supplementary material for: Imprinting defects at human 14q32 locus alters gene expression and is associated with the pathobiology of osteosarcoma
Source: Oncotarget. 2016 Jan 21;7(16):21298–314. doi: 10.18632/oncotarget.6965 (PMC5008286; doi:10.18632/oncotarget.6965)
Supplement: Supplementary file 1 [file oncotarget-07-21298-s001.pdf]

Imprinting defects at human 14q32 locus alters gene expression and is associated with the pathobiology of osteosarcoma

Supplementary Materials

Supplementary Table S1: Clinical information on normal bone tissues and OS patient samples

Normal bone tissues were collected from unrelated individuals who presented with diagnosis other than OS

Sample ID	Tumor tissue origin	Sex	Age	Remarks	14q-index
N1	Femur	F	20	none	4.5
N2	Femur	F	18	none	7.1
N3	Femur	M	15	none	11.0
N4	Femur	F	6	none	7.0
N5	Femur	M	13	none	5.0
N6	Femur	M	33	none	5.3
N8	Femur	F	15	none	9.0
N9	Rib	M	41	none	7.4

OS samples

Sample ID	Tissue	Sex	Age at Diagnosis	Chemo/ Radiation	Metastasis	14q-index
FT–8	Left distal femur	F	12	none	N	16.9
FT–14	Left distal femur	M	14	none	N	20.4
FT–429	Distal femur	M	16	none	N	6.9
FT–2	Mediastinum (bronchus)	F	16	none	Y	20.5
FT–7	Femur	M	16	Dox/Dex	N	10.7
FT–434	Lung	F	17	none	Y	17.3
FT–15	Left distal femur	M	17	none	N	16.2
FT–415	Hilar Lymph node	M	19	none	Y	26.5
FT–9	Left lung	M	19	none	Y	18.2
FT–10	Left lung	M	19	none	Y	26.9
FT–435	Chest wall mass	M	20	none	Y	30.7
FT–411	Lung	F	20	none	Y	5.9
FT–430	Lung	M	21	Chemo (n/a)	Y	29.8
FT–16	Left lung	M	22	none	Y	19.2
FT–424	Femur	M	24	none	N	12.3
FT–414	Chest	M	24	none	Y	25.5
FT–13	Right chest wall	M	24	none	Y	9.2
FT–413	Chest	M	*24/14	none	Y	27.6
FT–417	Knee	F	25	none	N	16.2
FT–11	Right knee	F	25	none	N	17.8
FT–422	Lung	F	*26/23	Chemo (n/a)	Y	17.3

FT–426	Lung	M	27	Study drug	Y	27.6
FT–428	Lung	M	27	none	Y	7.7
FT–17	Left lung	F	31	none	Y	7.3
FT–432	Lung	F	*31/29	none	Y	9.5
FT–5	Tibia	M	40	none	Y	6.6
FT–431	Forearm mass	M	42	none	N	14.2
FT–18	Right forearm	M	42	none	N	10.9
FT–425	Thigh	F	54	none	N	12.8
FT–427	Clavical mass	F	56	none	Y	5.1
FT–12	Right lung	M	57	none	Y	10.2
FT–419	Chest wall	M	65	none	Y	4.0

N = No; Y = Yes; *age at recurrence Dox = doxorubicin; Dex = Dexaroxane

ID	Age years	Sex	Subtype	Tumour Location	Huvos grade	Metastasis	ToM	ToD	ToF	14q-index
1	16.7	M	Telangiectatic	distal femur right	1	Y	0	25	25	15.7
2	17.1	F	Osteoblastic	distal femur left	1	N			147	8.0
3	25.3	M	Osteoblastic	proximal tibia left	2	Y	27	47	47	24.5
4	16.7	M	Osteoblastic	proximal humerus left	1	Y	0	11	11	13.5
5	16.5	M	Osteoblastic	proximal tibia left	3	Y	10	33	33	14.9
6	20.3	F	Anaplastic	distal femur right	3	N			94	28.4
7	18.6	M	Chondroblastic	proximal tibia left	3	Y	21	39	39	14.2
8	11.4	F	Osteoblastic	proximal tibia left	2	N			77	20.7
9	15.1	M	Osteoblastic	proximal tibia right	3	N			40	13.0
10	15.1	F	Osteoblastic	proximal tibia left	4	N			32	19.2
11	8.0	M	Osteoblastic	distal femur left	2	Y	0		31	15.5
12	8.6	M	Unknown	distal femur right	3	Y	2		31	18.5
13	13.7	M	Osteoblastic, some giant cells	distal femur right	3	N			24	22.4
14	13.8	F	Osteoblastic	distal femur right	3	N			24	15.6
15	9.5	F	Osteoblastic, some giant cells	proximal humerus left	3	N			17	7.7

ToM: time of metastasis (months)
ToD: time of death (months)
ToF: time of follow up (months)

Supplementary Table S2: Primer sequences used in this study

	**Bisulfite pyrosequencing**
**IG–DMR1**	
CG4–1st–F	ATGTTAATTATTTTTTGGATAAGAG
CG4–1st–R	AATCAAAACAACTCAAATCCTTTA
CG4–2nd–F	TTTTATTATTGAATTGGGTTTGTTAGT
CG4–2nd–RU	GGGACACCGCTGATCGTTTAAATCAAAACAACTCAAATCCTTTA
CG4–S	TGAATTGGGTTTGTTAGTA
**IG–DMR2**	
CG6–1st–F	GTTAAGAGTTTGTGGATTTGTGAGAAATG
CG6–1st–R	GTAAAAATGAGGAAAAGGGATAAAATGAG
CG6–2nd–FU	GGGACACCGCTGATCGTTTAAGTTGTTAGAGGTTTATAGTTGTTTAT
CG6–2nd–R	CATTATAACCAATTACAATACCACA
CG6–S	ATTACAATACCACAAAATTAC
**MEG–DMR**	
CG7–1st–F	TTGTGTTTGAATTTATTTTGTTT
CG7–1st–R	CCCCAAATTCTATAACAAATTACT
CG7–2nd–F	TGTTATAGAATTTGGGGGGTTTATTT
CG7–2nd–RU	GGGACACCGCTGATCGTTTACCCCAACTCTCCTAAAAAACC
CG7–S	GGTTAATTATTTTTAGAGAAATGA
H19–F1	TTTTTGGTAGGTATAGAGTT
H19–R	AAACCATAACACTAAAACCC
H19–F2	TAGTATATGGGTATTTTTGGAGG
H19–R2	GGGACACCGCTGATCGTTTAAAATATCCTATTCCCAAATAACC
H19–S	TGGTTGTAGTTGTGGAAT
MEST–F	TTGTTTATTTGAGGAGGGGGTGTTA
MEST–R	AATCTAAAAATCCTAACCCAAAAAAAT
MEST–RU	GGGACACCGCTGATCGTTTAAATCTAAAAATCCTAACCCAAAAAAAT
MEST–S	TTTGTTAGTAATGGAATGTTTAGAA
PEG3–F	GGTGTAGAAGTTTGGGTAGTTG
PEG3–R	CTCACCTCACCTCAATACTAC
PEG3–RU	GGGACACCGCTGATCGTTTCTCACCTCACCTCAATACTAC
PEG3–S	TGTTTATTTTGGGTTGGT
Universal–bio	Biotin-GGGACACCGCTGATCGTTTA

	**Real-time PCR primers**
MEG8–F	ACTCAAGCCCTTCATTCTGC
MEG8–R	AAGTCAGACCCAGGCAACAC
DIO3–F1	TGAGACTCCTGGGGAATGAC
DIO3–R1	ACACTCACCAAATGGCCTTC
DIO3AS–F	GGGAACGTCGAGACTGGAG
DIO3AS–R	GACAACGAAGAGCAGGAAGG
RTL1–F	AAGAGGCCATGTTCACCATC
RTL1–R	GCAGGATGAGCACAATCAGA
d3as–F1	AGCCGCGAAACTTCTTCTC
d3as–R1	CCTTCCAGCCTTGTCCAATA
TP53–F	GTTCCGAGAGCTGAATGAGG

TP53–R	TCTGAGTCAGGCCCTTCTGT
RB1–F	TCCCATGGATTCTGAATGTG
RB1–R	AGTTGGTCCTTCTCGGTCCT
DLK1–F	TTCACGGACTCTGTGGAGAA
DLK1–R	AGCATTCATAGAGGCCATCG
MEG3–F	ACGGCGGAGAGCAGAGAG
MEG3–R	ATGGAGAGGAGGTGGTCCTT
LINE–1–F	TTTTTTGAGTTAGGTGTGGG
LINE–1–RU	GGGACACCGCTGATCGTTTAGAAAGGGAATTTTTTGATTTT
LINE–1–S	AGTTAGGTGTGGGATATAGT

	**ChIP assays**
5kb–F	CGGAGACCAGCCTGACTAAC
5kb–R	TGGCTTACCTCAACCTCCAC
DLK1–CHIP–F	TCATAGTGCGCCTTTGTGTT
DLK1–CHIP–R	TTGGTACACGTTCCCTCACA
IG–DMR–CHIP–F	GAACTGGGTTTGCCAGTAGC
IG–DMR–CHIP–R	GTTGCAAACCAGGGTGAAGT
MEG–CHIP–F	TCCCTTCTTTGCTGCAATCT
MEG–CHIP–R	GGTTTATATGGAGGCGCAGA
RTL–CHIP–F	AGGCTGGCTCGATACAAGAA
RTL–CHIP–R	ACCGTGGAGATCAGAACCTG
SNO–CHIP–F	TCCAGTGATATGGGAGTGACA
SNO–CHIP–R	TACTTTCAAGGCCCGTGATT
CIT3–CHIP–F	AGTGGGCTGTCAATTTCAGG
CIT3–CHIP–R	AGGAAGGCCATTTCCTCTGT
DIO–CHIP–F	CCCTCAGCCCAAGATTTCTA
DIO–CHIP–R	CATCTGGGCTTCAGCAGTG
GAPDH–CHIP–F	CGGCTACTAGCGGTTTTACG
GAPDH–CHIP–R	AAGAAGATGCGGCTGACTGT
RARB–CHIP–F	AATCCTGGGAGTTGGTGATG
RARB–CHIP–R	TAGACCCTCCTGCCTCTGAA

Supplementary Table S3: DNA methylation levels of 12 Normal buccal samples and 13 OS family samples.

a) DMR-1 and DMR-2 DNA methylation analyses from 12 Normal buccal tissues

	DMR-1	DMR-2
Average	53.455	64.185
SD	2.467964	5.272018
95% CI	1.568069	3.34968
Upper	55.02307	67.53468
Lower	51.88693	60.83532

b) DMR-1 and DMR-2 DNA methylation levels of OS family samples

	Children		Mother		Father	
ID	DMR-1	DMR-2	DMR-1	DMR-2	DMR-1	DMR-2
22	80.46	75.75	80.69	81.76	75.27	85.81
30	71.18	76.38	74.16	75.73	79.62	77.95
72	68.58	83.21	66.21	75.17	66.57	67.48
85	59.92	73.13	73.82	78.39	72.4	74.87
48	67.31	70.83	71.27	67.07	61.87	63.37
57	64.61	78.09	65.2	66.46	65.39	67.51
91	72.49	71.58	58.35	69.96	76.64	72.24
42	65.14	79.8	68.42	61.72	70.68	72.78
41	64.84	65.27	72.1	70.12	79.39	78.69
94	61.53	63.58	64.97	53.17	65.45	72.85
2	73.63	62.08	70.88	65.39	77.27	73.85
51	62.08	58	68.19	51.09	64.96	62.98
83	63.2	61.53	61.83	60.73	67.3	63.61

Red color represent DNA methylation level increase, blue color represents DNA methylation level decrease.

c) Correlation between OS patient and their parent samples

DMR-1 Pearson r	OS patients	Mothers	Fathers
OS patients		0.3729379	0.5049415
Mothers	0.3729379		0.3392263
Fathers	0.5049415	0.3392263	

DMR-2 Pearson r	OS patients	Mothers	Fathers
OS patients		0.635883	0.2479064
Mothers	0.635883		0.6044092
Fathers	0.2479064	0.6044092	

Supplementary Table S4: DNA methylation levels of 12q DMR in Normal bone and Sleeping Beauty induced OS mice samples

a) DNA methylation levels of normal bone tissue of 4 normal mice

Sample ID	DNA methylation levels (%)
L1	73
L2	70
S3	59
S4	54
Average	64
SD	9
95% CI	9
Upper	73
Lower	55

b) DNA methylation levels of OS tissue

ID	Age (month)	Methylation (%)
46	8.5	69
47	10	56
48	8	59
58–1	9	62
58–2	9	65
60–1	11	73
60–2	11	76
62	10.5	63
63	14	88
67	12.25	79
84	14.5	92
87–1	11.33	83
87–2	11.33	53
87–3	11.33	60
87–4	11.33	73
87–5	11.33	75
88	9	80
91	13	71
103	12	58
113	11	64
142	8	51
165	10.5	49
229	9.25	69
237	11.5	56
273	8.5	83

Supplementary Table S5: DNA methylation levels of Normal bone and Rb/P53 mutation induced OS mice samples

a) DNA methylation levels of normal bone tissue of 4 normal mice

Normal	DMR methylation levels (%)
M–309	76
M–310	74
M–311	73
M–312	74

Average	74
SD	1
95% CI	2
Upper	76
Lower	72

b) DNA methylation levels of OS tissue

Sample ID	Note	Tumor	Mehylation (%)	Date
11	N69	left hind limb	25	2/4/2013
8	N72	left leg	44	11/9/2012
3	N60-L	left hind limb	49	10/30/2012
16	N93	left shoulder	49	4/8/2013
1	N32	right arm	50	12/15/2011
24	N105	left hind limb	50	6/27/2013
12	N102	right knee	54	2/20/2013
5	N58	left hind limb	55	10/30/2012
21	N91	right pelvis	58	4/12/2013
15	N93	right limb	59	4/8/2013
20	N91-1	left shoulder	60	4/12/2013
17	N86-R	right femur	62	4/8/2013
6	N60	soft tumor inside chest wall	64	10/30/2012
18	N91	left pelvis	67	4/12/2013
2	N50	big leg tumor	67	8/3/2012
10	N81	left pelvis	71	1/22/2013
19	N88	left forelimb	79	4/12/2013
22	N100	right hind leg	83	4/12/2013
9	N73	right shoulder	84	1/2/2013
4	N68	left uppter forelimb	87	10/30/2012
7	N72	right arm	88	11/9/2012
13	N92	neck tumor	88	3/13/2013
23	N96	right forelimb	91	5/23/2013
14	N86–L	left tibia	97	4/8/2013

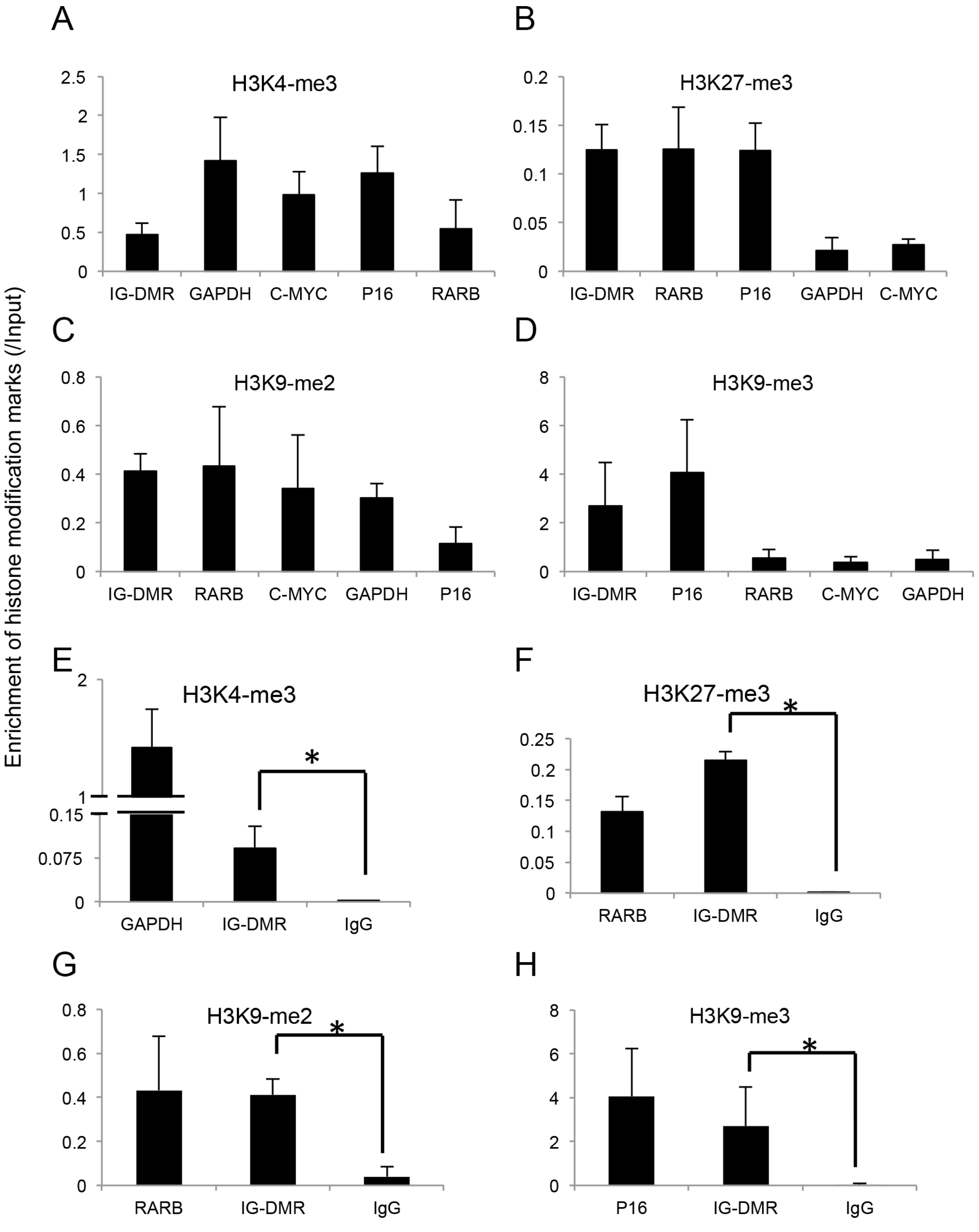

Supplementary Figure S1: Enrichment of H3K4-me3, H3K27-me3, H3K9-me2, and H3K9-me3 at IG-DMR. (**A–D**) Comparison of enrichment of H3K4-me3, H3K27-me3, H3K9-me2, and H3K9-me3 at IG-DMR to the promoter of GAPDH, C-MYC, P16, and RARB which were used as positive or negative controls for respectively. Input was used as internal control. (**E–H**) Comparison of enrichment of H3K4-me3, H3K27-me3, H3K9-me2, and H3K9-me3 to IgG, the negative antibody at 14q32 IG-DMR locus. *$P < 0.05$.

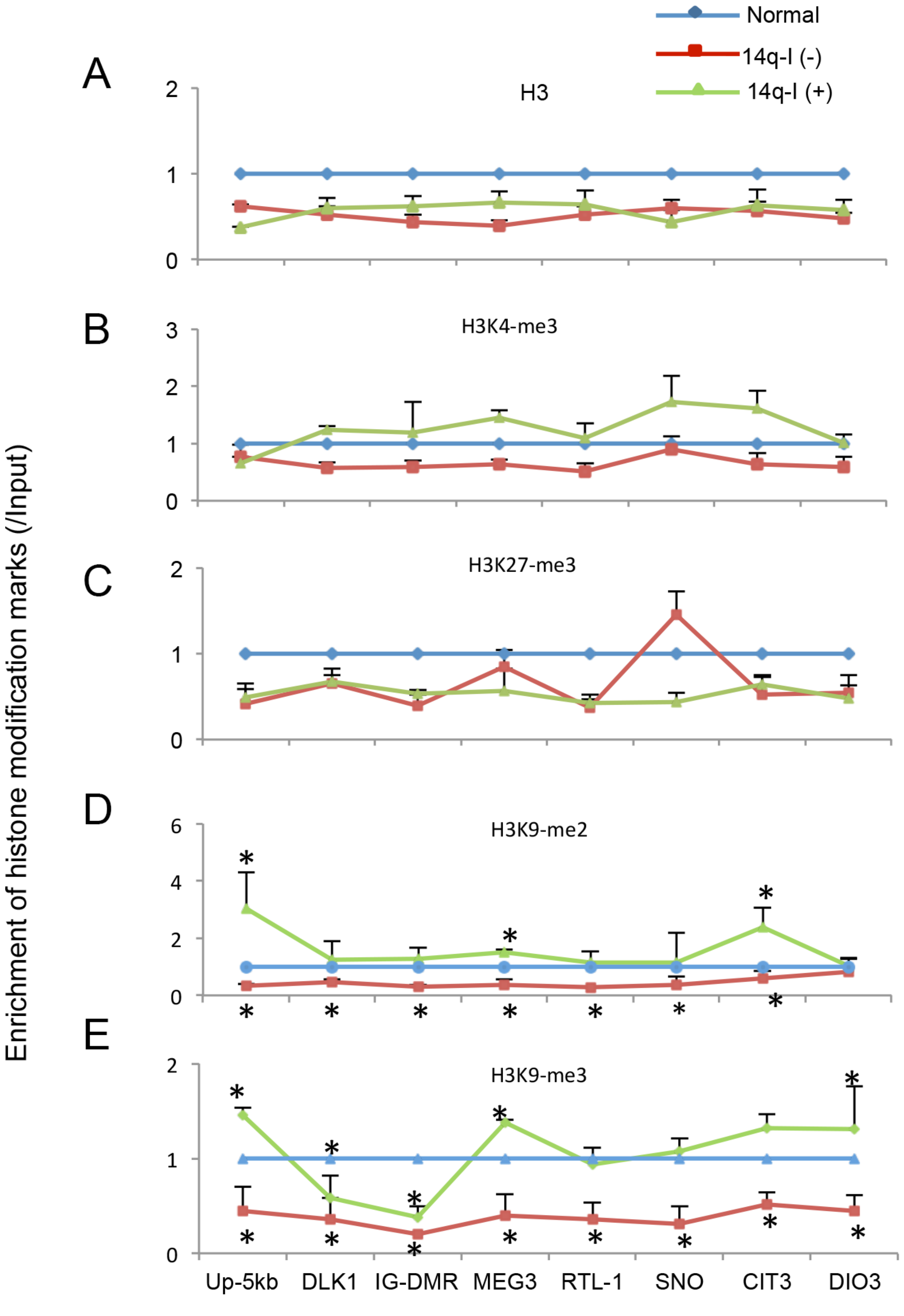

Supplementary Figure S2: Histone modification around the 14q32 imprinted region in normal and osteosarcoma tissues. Enrichment of H3, H3K4-me3, H3K27-me3, H3K9-me2, and H3K9-me3 around promoter regions of imprinted genes, IG-DMR and 5 kb upstream of *DLK1* transcription start sites. Input was used as internal controls. The values at IG-DMR site were used for normalization. Up-5kb: site ~ 5 kb upstream of the promoter of *DLK1*; SNO: small non coding RNA region; CIT: miRNA cluster 3 region.

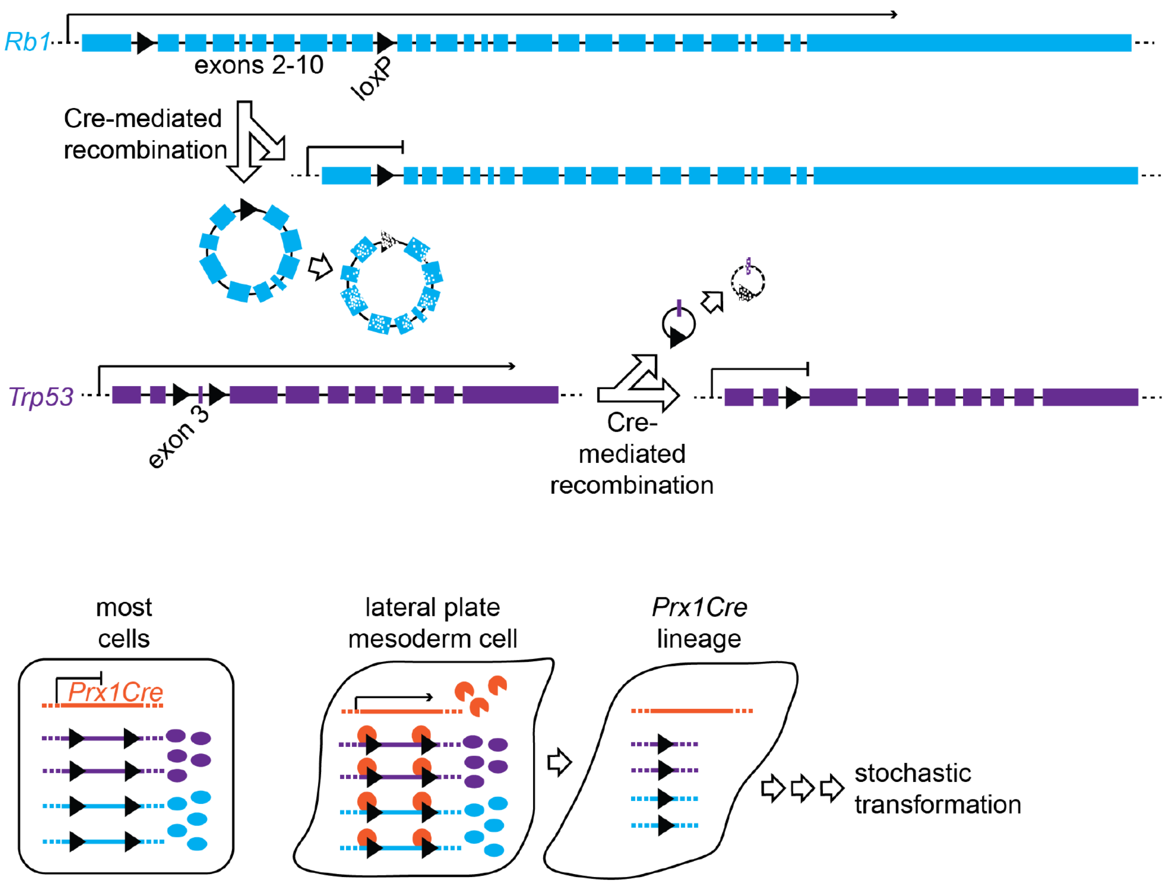

Supplementary Figure S3: Schematic for generation of spontaneous OS mouse model homozygous for conditionally disrupted alleles of Trp53 and Rb1.
